# Differential Progressive Remodeling of Coronary and Cerebral Arteries and Arterioles in an Aortic Coarctation Model of Hypertension

**DOI:** 10.3389/fphys.2012.00420

**Published:** 2012-11-12

**Authors:** H. N. Hayenga, J.-J. Hu, C. A. Meyer, E. Wilson, T. W. Hein, L. Kuo, J. D. Humphrey

**Affiliations:** ^1^Department of Biomedical Engineering, Texas A&M UniversityTX, USA; ^2^Department of Biomedical Engineering, National Cheng Kung UniversityTainan, Taiwan; ^3^Department of Systems Biology and Translational Medicine, Texas A&M Health Science CenterTX, USA; ^4^Department of Surgery, Texas A&M Health Science CenterTX, USA; ^5^Department of Biomedical Engineering, Yale University, New HavenCT, USA

**Keywords:** vascular smooth muscle cell, collagen, elastin, neointima, arterial remodeling, aorta, arteriolar function

## Abstract

**Objectives:** Effects of hypertension on arteries and arterioles often manifest first as a thickened wall, with associated changes in passive material properties (e.g., stiffness) or function (e.g., cellular phenotype, synthesis and removal rates, and vasomotor responsiveness). Less is known, however, regarding the relative evolution of such changes in vessels from different vascular beds. **Methods:** We used an aortic coarctation model of hypertension in the mini-pig to elucidate spatiotemporal changes in geometry and wall composition (including layer-specific thicknesses as well as presence of collagen, elastin, smooth muscle, endothelial, macrophage, and hematopoietic cells) in three different arterial beds, specifically aortic, cerebral, and coronary, and vasodilator function in two different arteriolar beds, the cerebral and coronary. **Results:** Marked geometric and structural changes occurred in the thoracic aorta and left anterior descending coronary artery within 2 weeks of the establishment of hypertension and continued to increase over the 8-week study period. In contrast, no significant changes were observed in the middle cerebral arteries from the same animals. Consistent with these differential findings at the arterial level, we also found a diminished nitric oxide-mediated dilation to adenosine at 8 weeks of hypertension in coronary arterioles, but not cerebral arterioles. **Conclusion:** These findings, coupled with the observation that temporal changes in wall constituents and the presence of macrophages differed significantly between the thoracic aorta and coronary arteries, confirm a strong differential progressive remodeling within different vascular beds. Taken together, these results suggest a spatiotemporal progression of vascular remodeling, beginning first in large elastic arteries and delayed in distal vessels.

## Introduction

Hypertension affects up to a third of the adult population in the United States and is a significant risk factor for many diseases, including aortic aneurysms and dissections, atherosclerosis, end stage renal failure, heart failure, intracranial aneurysms, and stroke. Hypertension is caused by and causes significant remodeling of arteries and arterioles, which results from phenotypic changes by vascular cells and associated changes in extracellular matrix that often lead to an increased stiffness of the vessel and dysfunctional behaviors. Indeed, mounting evidence suggests that stiffening of central arteries (e.g., the aorta) may be both an initiator and an early indicator of subsequent cardiovascular risk, particularly for low resistance organs such as the brain and kidneys (O’Rourke and Nichols, 2005; Payne and Webb, 2006; Greenwald, 2007; Narkiewicz et al., 2007; Glasser and Arnett, 2008). There is a pressing need, however, to understand better the possible spatial as well as temporal progression of vascular stiffening and dysfunction in hypertension, including causes of the increases in large artery stiffness that can, in turn, increase pulse wave propagation and exacerbate the hypertension by affecting arteriolar function in vital organs (Davidson et al., 1985; Humphrey and Na, 2002; Garcia and Kassab, 2009). In particular, impaired vasodilation has been reported with hypertension due to elevated mean arterial pressure (MAP; Miller et al., 1987) or pulse pressure (Ceravolo et al., 2003); it is associated with an imbalance between the generation and release of vasodilator and vasoconstrictor compounds due to endothelial dysfunction (Tang and Vanhoutte, 2010). Decreased production or availability of the potent vasodilator nitric oxide (NO) from the endothelium is thought, for example, to contribute to a decreased response to adenosine (Zhang et al., 2004), an endogenous metabolite involved in the control of local blood flow. In addition to regulating vasodilation, NO and other vasodilator compounds also inhibit the expression of collagen and smooth muscle growth that affect vascular stiffening, while endothelium-derived vasoconstrictors increase these processes (Rizvi et al., 1996; Rizvi and Myers, 1997).

The goal of this work, therefore, was to use an established aortic coarctation model of hypertension in the mini-pig to contrast, for the first time, progressive morphological and histological changes in the proximal descending thoracic aorta, coronary artery, and cerebral artery as well as possible changes in arteriolar function in the heart and brain to document differences in the type, extent, and time course of vascular remodeling in multiple vascular beds within a single animal. Such information is vital for an increased understanding of the development of hypertension as well as to enable a data-driven development of computational models of vascular remodeling in hypertension (Gleason and Humphrey, 2004; Valentin et al., 2009).

## Materials and Methods

### Animal model

All animal protocols were approved by the Institutional Animal Care and Use Committee at Texas A&M University. Selected arteries and arterioles were excised from adult mini-pigs (Panepinto Micro Mini pigs; Mansonville, CO, USA) before or at prescribed times following the surgical creation of a supra-diaphragmatic aortic coarctation that induced hypertensive (HT) conditions in the vessels of interest (Fossum et al., 2003). Briefly, following isoflurane-induced anesthesia, a balloon-inflatable silicone occluder (*In vivo* Metrics, Inc.; reinforced by Solomon Scientific), prefilled with a 50% dextrose solution, was placed over a Gore-Tex patch that was wrapped around the supra-diaphragmatic aorta, between intercostal vessels, and secured with suture. The occluder was connected via stiff tubing to a vascular access port placed subcutaneously in the neck, which allowed it to be inflated or deflated within the conscious animal based directly on desired changes in blood pressure that were measured via an indwelling transducer placed within the internal thoracic artery. Arterial pressure and heart rate were recorded via telemetry (Data Sciences Inc., St. Paul, MN, USA) for 30 s every 2 h throughout the study.

Beginning approximately 1 week after surgery, the aorta was gradually coarcted by adding small amounts of dextrose to the occluder over a 7–10-day period until the daily average MAP reached or exceeded 150 mmHg. Data were collected from 41 mature (7–16 months old) male mini-pigs (Sinclair Research Center, Inc., Columbia, MO, USA): 16 normotensive (NT) controls and 25 HT animals. Specifically, vessels were harvested from true controls (*n* = 2), surgery controls (*n* = 3) without an occluder, NT animals at 2 (*n* = 2), 4 (*n* = 4), 6 (*n* = 3), and 8 (*n* = 2) weeks following a sham surgery wherein an occluder was implanted but not inflated, and from HT animals at 2 (*n* = 7), 4 (*n* = 7), 6 (*n* = 5), and 8 (*n* = 6) weeks after the animal reached its target MAP (>150 mmHg). Note that MAP did not differ amongst the true and surgery control NT groups over the 8-week period of study (ANOVA, *p* > 0.05). In contrast, the sham NT controls had a 16 mmHg increase in MAP, suggesting that the Gor-Tex graft and/or occluder may have raised the aortic pressure despite not being inflated (Fossum et al., 2003). Using a larger occluder may have prevented this problem. Therefore, the sham NT arterial sections are not reported herein due to inconsistent results with some displaying significant thickening. Instead “NT controls” or “week 0” refers to only the true and surgery controls. Notwithstanding increases in mean and pulse pressure over time during hypertension (see Table A1 in Appendix, which lists the average MAP, pulse pressure, and pulse rate at 0, 2, 4, 6, and 8 weeks of HT), the overall averaged MAPs were 131 ± 6 mmHg in NT and 168 ± 17 mmHg in HT animals, which were significantly different (*p* < 0.001); associated overall averaged pulse pressures were 37 ± 6 mmHg in NT and 53 ± 10 mmHg in HT animals, which were also significantly different (*p* < 0.001). The overall statistically different averaged pulse rates were 34 ± 4 bpm in the NT and 52 ± 10 bpm in the HT animals (*p* < 0.01). Finally, the mean age of the mini-pigs was not statistically different between the NT and HT groups (*p* > 0.05).

### Histology and immunohistochemistry

Immediately following exsanguination, segments of descending thoracic aorta 3 cm proximal to the occluder (P-Ao) as well as segments of the left anterior descending coronary artery (LAD) and the middle cerebral artery (MCA) were dissected free of perivascular tissue and fixed under unloaded conditions by immersion in fresh 4% paraformaldehyde for 1 h. Specimens were then embedded in paraffin, sectioned at 5 μm increments, deparaffinized, rehydrated, and stained for either standard histology or immunohistochemistry. Prior to immunostaining, slides were placed in a local greenhouse for 5 days to reduce autofluorescence of endogenous fluorophores, primarily from elastin and collagen (Kingsley et al., 2001; Monici, 2005). Sections were stained with Verhoeff–van Gieson (VVG) for elastin (Verhoeff) and collagen (van Gieson), picro-sirius red (PSR) for fibrillar collagens, and with antibodies against alpha smooth muscle actin (αSMA) for contractile cells, von Willebrand factor (vWF) for endothelial cells of the cerebral arteries, MAC387 for leukocytes, and CD34 for hematopoietic progenitor stem cells. Antibody specifications and optimal dilutions are detailed in Table A2 in Appendix.

Indirect immunofluorescent staining was performed following the Abcam double immunofluorescence protocol (Abcam, 2010) with slight modifications. Optimal antigen retrieval involved heat-induced epitope retrieval (using a pressure cooker) for 3 min with a sodium citrate retrieval buffer at pH = 6 for all antibodies. Sections with intracellular target proteins (i.e., αSMA, vWF, and MAC387) were permeabilized with 0.25% Triton X-100 in PBS for 10 min. Subsequently, slides were incubated in 10% goat serum for 30 min to prevent non-specific binding of antibodies. Slides were incubated with the primary antibody, washed 3x for 5 min in PBS, and incubated with their fluorescent secondary antibodies (see Table A2 in Appendix, which specifies antibody concentration, time, and vendors). Slides were then either incubated for 30 min with fluorescent tertiary antibodies for greater fluorescence, as in the case of the low-abundance target proteins MAC387 and CD34, or washed 3x for 5 min in PBS and counter-stained with premixed DAPI mounting medium [ProLong^®^ Gold antifade reagent with DAPI, Invitrogen (P-36931)]. After tertiary incubation for MAC387 and CD34, sections were mounted with the premixed DAPI medium and cover-slipped for imaging. All experiments were performed with a negative control (i.e., primary antibody substitution with PBS) to determine background fluorescence. A negative isotype control [rabbit IgG, Vector Laboratories (I-1000)] was also used to confirm specific staining.

### Image acquisition and quantification

Arterial samples were imaged using an Olympus BX/51 fluorescence microscope coupled with an Olympus DP70 digital camera. This microscope is equipped with diascopic (transmitted) and episcopic (reflective) pathways; it is also customized for circularly polarized illumination (Hu et al., 2006) to capture polarized light images of PSR-stained sections. Köhler illumination was established and settings for microscopic illumination, exposure times, and color balance of the digital camera were fixed for each set of slides to enable subsequent comparisons. For fluorescence images, settings were found by first zeroing out the fluorescence in the negative control for that artery-type (i.e., a section processed exactly the same except without the primary antibody) for each batch of staining.

For quantitative morphological analyses, an appropriate objective was used to acquire an image of the entire cross-section (i.e., dissection microscope for P-Ao, 4X for LAD, and 20X for MCA) as well as at least two representative higher magnification images including a transmural section of the arterial wall (i.e., 4X for P-Ao, 20X for LAD, and 40X for MCA) per specimen. In a few cases, images were acquired at an even higher magnification for better visualization. Image files were saved in tagged-image file format (TIFF). GIMPshop 2.2.8 was used to overlay fluorescent images taken with different filters (i.e., Fitc, Tritc, PI).

A “detail analysis” was used to determine the following for each artery: (1) inner and outer radius, (2) mean wall thickness, (3) mean thickness of the intimal, medial, and adventitial layers, (4) area fraction of positive αSMA stain in the wall, (5) area fraction of fibrillar collagen in the wall, (6) area fraction of elastin in the wall, (7) nuclei count per wall area, (8) area fraction of hematopoietic cells, (9) area fraction of macrophages, and (10) endothelial cells in the cerebral arteries. Specifically, mean inner and outer radii and thicknesses were determined from VVG or PSR-stained sections for the three different types of arteries from all 41 pigs based on images of whole arterial rings. Whereas loose perivascular tissue was physically dissected from the P-Ao before sectioning, we used ImageJ and GIMPshop to digitally remove (crop) the myocardium and connective tissue from the adventitia of the LAD and MCA. The luminal boarder was detected using magic wand in GIMPshop, with the lumen filled black for better image recognition. An image analysis program was written in MATLAB 7.9.0 (R2009b) to calculate the luminal and wall area as well as the luminal and outer adventitial perimeter. Formulas for the area of a circle, circumference, and hydraulic diameter were used to calculate radius and thickness for each vessel. The thickness calculations were then verified using the images taken at a greater magnification of a transmural section of the arterial wall. The connective tissue was again cropped from the adventitia for the LAD and MCA. To obtain layer-specific thicknesses, the intima and/or media were cropped separately. The region of interest was found using a color threshold module and refined using a median filter (to remove small particles). The mean thickness was then calculated by dividing the total area (pixels^2^) by the image width (pixels) and then using the scale bar to convert to metric units. That is, for an image having dimensions *m *× *n*, where *m* is the width and *n* is the height, the mean thickness was defined as $\bar{\text{thickness}}=\sum_{i=1}^{m}n_{i}^{\text{Wall}}/m$, where, $n_{i}^{\text{Wall}}$ is the thickness of the wall at each pixel (*i*) along the image. Intimal thickness was found by subtracting medial and adventitial thicknesses from the total. Similarly, to determine the distribution of nuclei through the wall (from lumen to adventitia), a MATLAB routine was written to find the centroid of each nuclei from fluorescence images. The number of nuclei as a function of wall position (where 0 is the lumen and 1 is the outer adventitia boarder) was determined for each image. Then, the number of nuclei was normalized by dividing the wall thickness (*n*) and width (*m*) into 25 segments each and dividing by the largest number of nuclei in the *n* direction.

To quantify area fractions of positive fluorescence (for αSMA, CD34, and MAC387), we first cropped the lumen and connective tissue, then filled the wall with white for easy recognition. Second, images were converted to grayscale for each channel (red, green, blue), with pixel values ranging from 0 to 255 depending on the intensity of a specific color. Third, we determined the proper threshold value within the range (0–255) to convert the red, green, or blue channel images into a binary image. To do this, we subtracted the total color-specific intensities in the arterial wall from the color-specific intensities in a negative control (i.e., a mostly black image of the same sample with no primary antibody). The threshold cut-off was set to the minimum value for the last bin in the resulting mode. This was done for each set of images as a batch process (divided based on the microscope objective used and the stain), thereby ensuring specific positive staining with minimal background or observer bias. Lastly, the total pixels identified according to the stain were then divided by the total pixels constituting the arterial wall to yield the area fraction of positive stain in the wall section.

**Figure A1:**
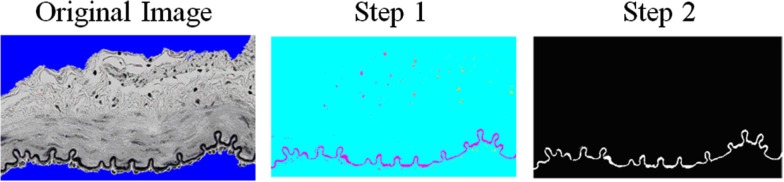
**Algorithm created to determine the area fraction of elastin in the wall**. First, a threshold was used to determine the “black” elastin from the VVG stained images. Second, a threshold was added to remove the black nuclei blobs. Then, the ratio of elastin pixels to wall pixels (non-blue portion) was determined.

A custom routine was created in MATLAB to analyze samples stained for elastin using VVG. The VVG stain also stains nuclei in some cases (see Figure A1 in Appendix, which illustrates nuclei detection and removal), thus the program included an adaptable threshold designating the minimum size of a recognized blob (i.e., nuclei). Autofluorescence was not an accurate measure for elastin because we placed the samples in a greenhouse for 5 days to reduce the natural autofluorescence. In addition, for the large aortic samples, we had to use a 4X objective and thus lost much of the weak autofluorescent signal.

The area fraction of total fibrillar collagen was quantified similarly from PSR birefringent images. Briefly, the portion stained red under non-polarized light was used to determine the region of interest and the same field under polarized light was used to quantify the collagen (birefringence), with anything not black by a threshold level considered to be collagen. The collagen area (in pixels) was then divided by the total wall area (in pixels) to get the proportion of collagen in the wall. Finally, the total number of cells was determined by a custom program for nuclei written in MATLAB. The centroid of each nucleus was identified according to minimum size, separation, and intensity thresholds. In addition, inner and outer boundaries of the vessel wall were found based on orientation and color threshold (since the extravascular areas were cropped to white). The number of nuclei relative to wall position, with 0 being the luminal boundary and 1 being the adventitial boundary, was calculated for each artery.

### Arteriole function testing

Single coronary and cerebral arterioles (60–100 μm in internal diameter *in situ*, 0.6–1.0 mm in length without branches) were isolated from the surface of the left ventricular subepicardium and cerebral cortex, respectively, for functional study as described previously (Kuo et al., 1991). These vessels were then cannulated with micropipettes filled with PSS (in mM, NaCl 145.0, KCl 4.7, CaCl_2_ 2.0, MgSO_4_ 1.17, NaH_2_PO_4_ 1.2, glucose 5.0, pyruvate 2.0, EDTA 0.02, MOPS 3.0, and 1% bovine serum albumin) and placed in a Lucite vessel chamber that was transferred to the stage of an inverted microscope (model IM35, Zeiss, Thornwood, NY, USA). These vessels were pressurized without flow at 44.1 mmHg by an adjustable reservoir system and allowed to equilibrate in PSS at 36–37°C to allow basal tone to develop. Internal diameter was measured throughout the experiment using video microscopic techniques, also as described previously (Kuo et al., 1991).

Possible effects of hypertension (at 8 weeks) on vasomotor responses were assessed via exposure to adenosine (0.1 nmol–10 μmol/l; Sigma, St. Louis, MO, USA), a known endothelium-dependent NO mediated vasodilator for porcine coronary (Hein and Kuo, 1999; Hein et al., 1999) and cerebral (Armstead, 1997) arterioles. The contribution of NO was confirmed by treating the vessels with the specific NO synthase (NOS) inhibitor N^G^-nitro-l-arginine methyl ester (l-NAME, 10 μmol/l) for 30 min (Hein and Kuo, 1999; Hein et al., 1999). The vessels were exposed to each concentration of adenosine for 3–5 min to establish a stable diameter. At the end of each experiment, the vessels were relaxed with sodium nitroprusside (0.1 mmol/l) to obtain the maximal diameter at 44.1 mmHg intraluminal pressure. All diameter changes in response to adenosine were normalized to the vasodilation in response to sodium nitroprusside and thus expressed as a percentage of maximal dilation.

### Statistics

All data are presented as mean ± SEM. Statistical comparisons for all data were performed as appropriate with Student’s *t*-test or two-way ANOVA with or without repeated-measures and tested with a Bonferroni multiple range test. Results were considered significant if: *0.01 < *p* < 0.05, **0.001 < *p* < 0.01, or ***p < 0.001.

## Results

**Figure 1:**
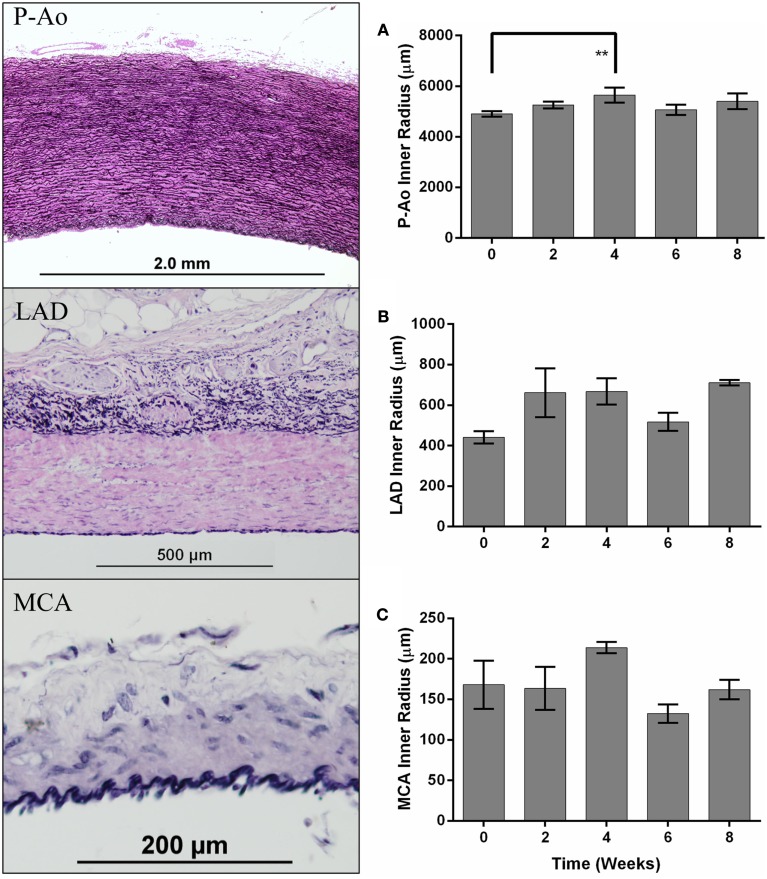
**Differences in arterial structure and quantification of inner radii over time**. The panel on the left shows representative histological images of the descending thoracic aorta, just proximal to the site of the balloon occluder (P-Ao), the left anterior descending (LAD) coronary artery, and the middle cerebral artery (MCA) from a control normotensive mini-pig stained with Verhoeff–Van Gieson (VVG) for elastin in black and collagen/smooth muscle in pink. Inner radius of control (week 0) and hypertensive arteries (weeks 2, 4, 6, and 8), mean ± SEM, for P-Ao **(A)**, LAD **(B)**, and MCA **(C)**. The inner radius was calculated using the circle formula based on the wall area calculated from images showing the entire artery. **0.001 < *p* < 0.01, two-way ANOVA with Bonferroni *post hoc* analysis.

**Figure 2:**
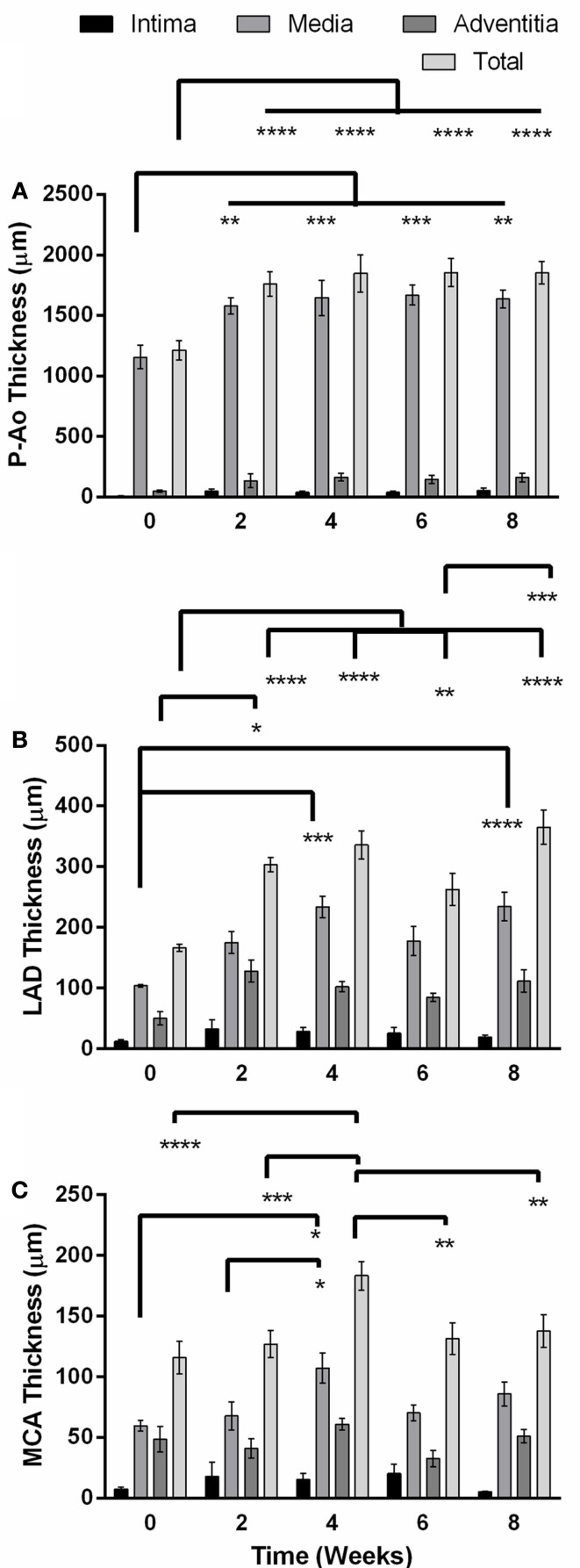
**Unloaded total wall thickness (mean ± SEM) and its distribution amongst the intima, media, and adventitia in the descending thoracic aorta just proximal to the balloon occluder (P-Ao), the left anterior descending artery (LAD), and the middle cerebral artery (MCA) before creation of the coarctation (week 0) and at 2, 4, 6, and 8 weeks thereafter**. **(A)** The P-Ao thickened significantly in the media at and after 2 weeks. **(B)** The LAD thickened significantly via both the adventitia at 2 weeks, and media at 4 and 8 weeks. **(C)** The changes in the MCA at week 4 may have been anomalous. *0.01 < *p* < 0.05, **0.001 < *p* < 0.01, ***p < 0.001, two-way ANOVA with Bonferroni *post hoc* analysis.

The aorta is classified as an elastic artery due to its many concentric elastic lamellae; the coronary and cerebral arteries have only two and one elastic lamellae, respectively, and are thus classified as muscular arteries. A sustained MAP above 150 mmHg for up to 8 weeks led to significant alterations in geometry of the P-Ao and LAD, but not the MCA, hence remodeling did not depend on arterial classification alone. Figure 1 shows gross structural differences amongst the NT P-Ao, LAD, and MCA, including the number of elastic lamellae, total wall thickness, and spatial distribution of elastin (black) and SMCs (light pink). Unloaded inner radius did not change significantly for the LAD or MCA over the 2, 4, 6, or 8-weeks of hypertension (Figures 1B,C), though it did increase in the LAD, on average, 50, 51, 17, and 61% at 2, 4, 6, and 8 weeks, respectively, compared to the NT controls (Figure 1B). The unloaded inner radius of the P-Ao also did not change significantly over this period with the exception of a 17% increase at 4 weeks of hypertension. In contrast, the wall thickened significantly in the P-Ao and LAD over the 2, 4, 6, and 8-weeks (Figure 2, darkest bar). For example, mean aortic wall thickness, as calculated from the measured area of the entire ring, increased significantly (by 56%) after only 2 weeks of hypertension and remained thicker (64%) after 8 weeks. The increase in wall thickness was even more drastic in the LAD. Mean wall thickness increased 220% after 2 weeks of hypertension and continued to be greater at 4 (by 250%) and 8 (by 242%) weeks. Results at 6 weeks also showed thickening, but not as drastic; the reason for this slight decrease at 6 weeks was not clear, but other issues arose at the 6-weeks time point as well. The same trend, but slightly less drastic, was also observed using the thickness calculated from selected transmural images. The data suggested that the MCA increased in wall thickness at weeks 4 of hypertension, but this may have been anomalous given the lack of change at 2, 6, and 8 weeks (Figure 2, darkest bar).

Layer-specific distributions for the three layers of the arteries (i.e., intima, media, and adventitia) revealed that thickening of the P-Ao was due almost entirely to increases in the medial layer and to a lesser extent the intimal and adventitial layers (although not significant; Figure 2A). Likewise, the LAD thickened primarily via increases in the medial layer. The adventitial layer exhibited a threefold increase after only 2 weeks (Figure 2) and stayed elevated thereafter, but not significantly. By 4 weeks, however, the medial layer had increased in thickness by 2.2-fold (significantly) while the adventitial layer was still 2.5-fold thicker (non-significantly) than the NT controls. Consistent with the findings for the average overall thickness of MCAs at each time point, the average thickness for each layer did not change significantly over time, except for the media at 4 weeks.

Despite significant changes in wall thickness in the P-Ao and LAD, cell density (i.e., cells per square micrometer) remained nearly constant over the 8 weeks, thus suggesting proportional increases in cell number with thickness. It is noted, however, that the initial cell densities depended on location: 0.000127, 0.00378, and 0.005497 cells/μm^2^ for the P-Ao, LAD, and MCA, respectively. Thus, relatively speaking, cell density increased while the amount of elastin in these arteries decreased. A significant decrease (15%) in cell density was observed at week 6 in the MCA, but this too may have been anomalous since the average value was recovered by week 8.

**Figure A2:**
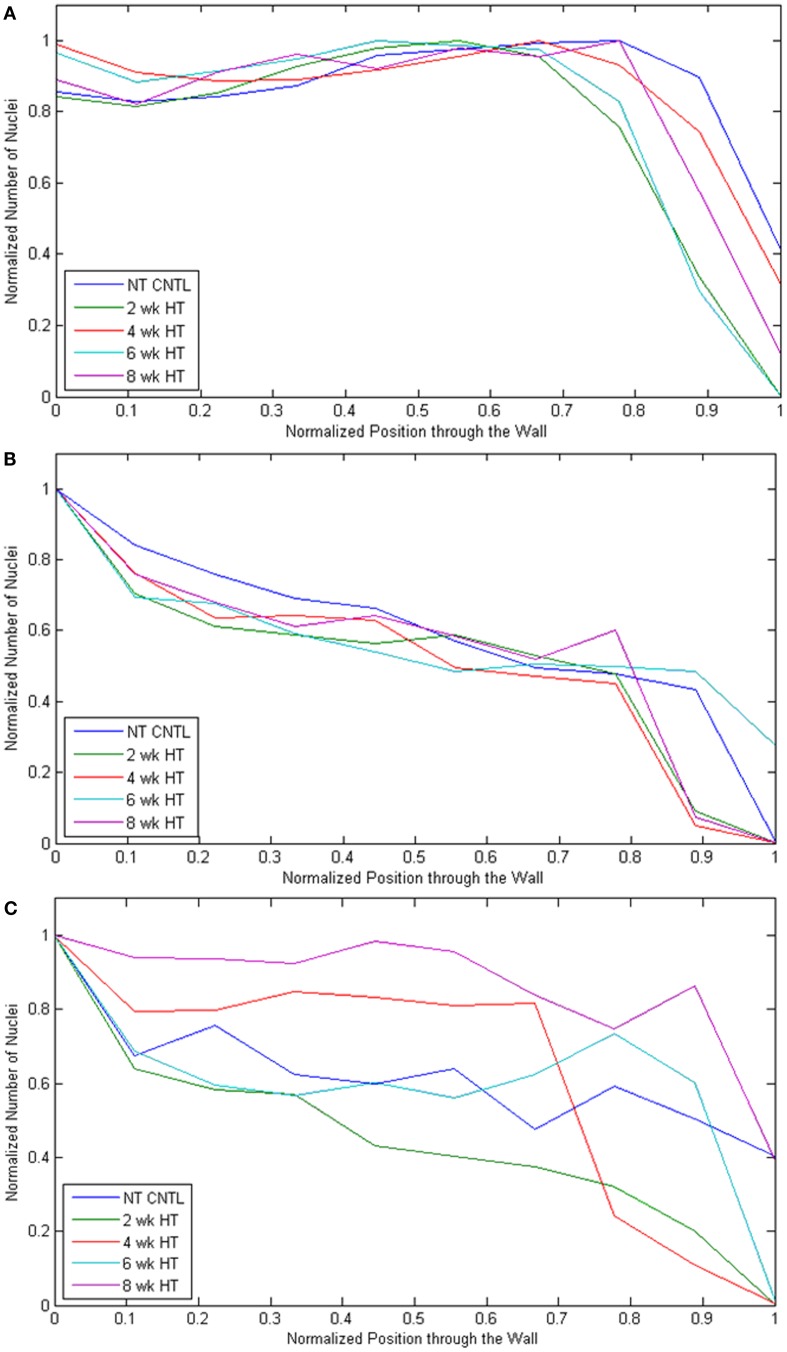
**The number of nuclei were normalized compared to the most dense segment in the radial direction and plotted as a function through the arterial wall going from lumen “0” to adventitia “1” for each artery type [(A) P-Ao, (B) LAD, (C) MCA] at each experimental time point (NT control, and 2, 4, 6, and 8 weeks HT)**.

The number of nuclei was also quantified as a function of radial position from the lumen to the outer adventitial border. Notably, both the P-Ao and LAD maintained similar distributions throughout the period of growth and remodeling (see Figures A2A,B in Appendix, which illustrates nuclei density over time for each artery and condition). Although the MCA changed the least in regards to geometry, cell phenotypic changes, and matrix production/removal, the radial distribution of nuclei varied the most over time (0–8 weeks) in the MCA (see Figure A2C in Appendix, which shows MCA nuclei density over time). Nevertheless, cell density was highest within the inner 10% of the wall in all three types of arteries. Cell density was spatially the most uniform in the P-Ao, being nearly constant until the outer ∼25% of the vessel (presumably the adventitia; Figure A2A in Appendix).

**Figure 3:**
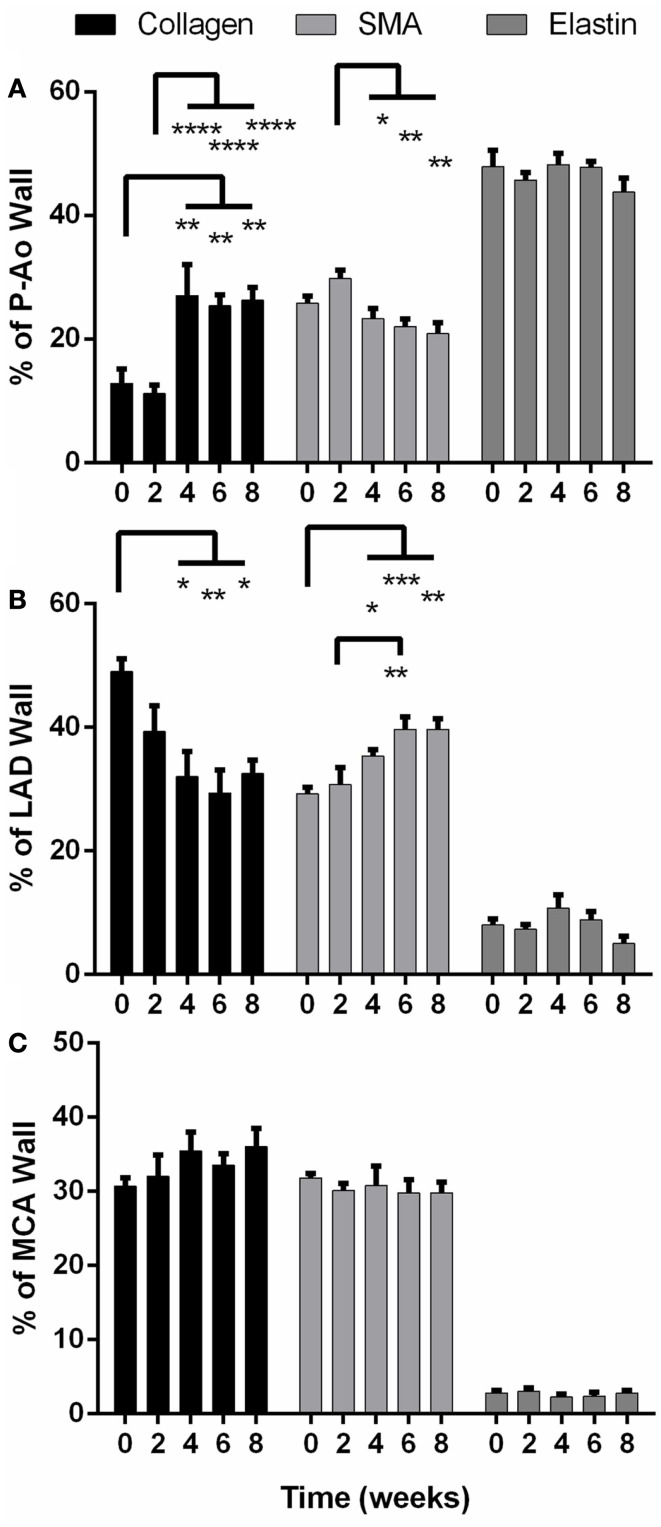
**Percentage of structurally significant constituents (collagen, smooth muscle, and elastin) in the arterial wall of the proximal aorta (P-Ao), left anterior descending artery, (LAD), and middle cerebral artery (MCA), mean ± SEM**. **(A)** Collagen increased significantly in P-Ao at and after 4 weeks; SMA increased at week 2 (ns) and proceeded to decrease significantly compared to week 2. **(B)** In the LAD, collagen was significantly decreased at and after 4 weeks while smooth muscle was significantly increased. **(C)** These constituents did not change significantly in the MCA. *0.01 < *p* < 0.05, **0.001 < *p* < 0.01, ***p < 0.001, two-way ANOVA with Bonferroni *post hoc* analysis.

Birefringence in PSR-stained sections suggested that the basal value of fibrillar collagen in the P-Ao constituted only about 13% of the arterial wall (Figure 3A). Collagen was not significantly different after 2 weeks of hypertension, but dramatically increased twofold at 4 weeks and stayed consistently elevated for 6 and 8 weeks. Conversely, the percentage of the wall of the LAD occupied by fibrillar collagen decreased significantly from 49 to 32% by week 4 consistent with an increase in smooth muscle (Figure 3B). Unlike the P-Ao and LAD, collagen did not change over the 8-weeks period in the MCA (Figure 3C).

Smooth muscle alpha actin is a marker of smooth muscle cells exhibiting a contractile phenotype. The percentage of αSMA increased by 16% (albeit not statistically significant) in the P-Ao after 2 weeks of hypertension (Figure 3A), after which smooth muscle contractility decreased slowly (and significantly compared to week 2). There was 19% less αSMA by 8 weeks of hypertension when compared to the NT controls (not significant). After 2 weeks, the LAD exhibited the opposite effect (Figure 3B): there was a sustained increase in overall αSMA and by inference, wall contractility. That is, from 4 to 8 weeks, the percentage of αSMA was approximately 40% higher than control. The distribution of constituents – elastin, fibrillar collagens, and αSMA – did not change significantly in the MCA (Figure 3C).

Under normal conditions, functional arterial elastin is thought to be minimally produced after the perinatal period (Shapiro et al., 1991; Davis, 1993; Langille, 1996; Humphrey, 2002). Nevertheless, changes in the percentage of other constituents can change the area or mass fractions of elastin. Herein, however, we found that the relative content of elastin remained nearly the same in all arteries over the 8-weeks of hypertension. Although not shown, the elastin in the internal elastic lamellae appeared straighter in the unloaded configuration in both the LAD and the MCA than in the P-Ao.

**Figure 4:**
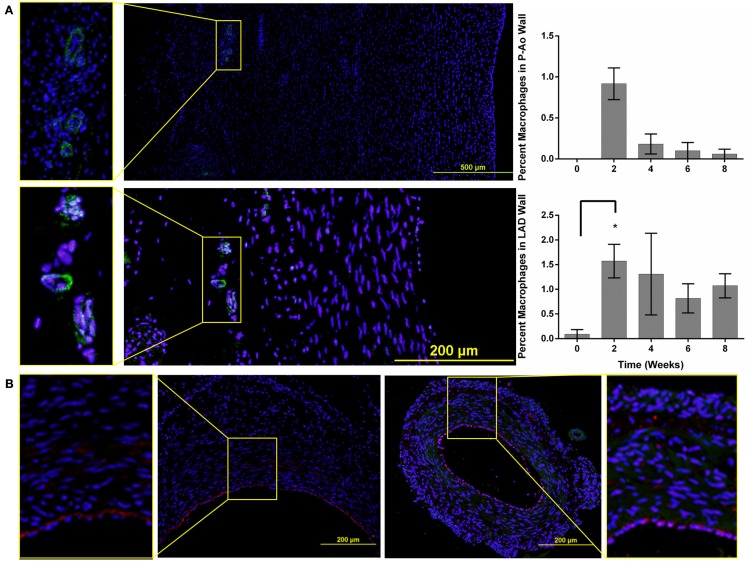
**Macrophages and CD34 positive cells are seen in all three artery types**. **(A)** Representative images of the P-Ao (top) and LAD (bottom) at 4 weeks of hypertension. The zoomed-in images to the left highlight macrophages observed in the adventitia. The percentage of macrophage present was determined as the ratio of the positively stained area compared to the total arterial area. In the P-Ao, macrophage involvement was greatest in the adventitia near the vasa vasorum at week 2. In the LAD, relative macrophage content was greater at week 2 and remained elevated at weeks 6 and 8 although not significantly. **(B)** Representative images of CD34 stained (red), LAD (left), and MCA (to the right) for the normotensive controls. The zoomed-in images to the left and right highlight the presence of CD34 positive cells in the intima and media of both arteries. *0.01 < *p* < 0.05, two-way ANOVA with Bonferroni *post hoc* analysis.

In addition to alterations in extracellular matrix and cellular phenotype, circulating blood cells can also play key roles in arterial remodeling due to elevated blood pressure. Indeed, progenitor cells are also present in normal arteries (Hu et al., 2004; Torsney et al., 2005; Passman et al., 2008). Throughout the 8-weeks time course of hypertension, the amount of CD34 staining did not change significantly in the P-Ao or the LAD relative to control. The average value was 1.3 and 0.9% of the wall for the P-Ao and LAD, respectively. Interestingly, however, the percentage of CD34 staining increased from 1 ± 0.7 to 1.6 ± 0.4 in the MCA by week 4 and remained elevated (although not significant). Unlike the progenitor cells, essentially zero macrophages were present in all of the control arteries. Macrophages were similarly not observed in any of the MCA arteries after the onset of hypertension. In contrast, there was an increase in MAC387 in both the P-Ao and the LAD by 2 weeks of hypertension (Figure 4A); these macrophages were seen in the both media and the adventitia but this observation was too inconsistent to be statistically significant for the P-Ao. Whereas the number of macrophages decreased dramatically and monotonically in the P-Ao, it remained elevated in the LAD over the 8-weeks (Figure 4A).

**Figure 5:**
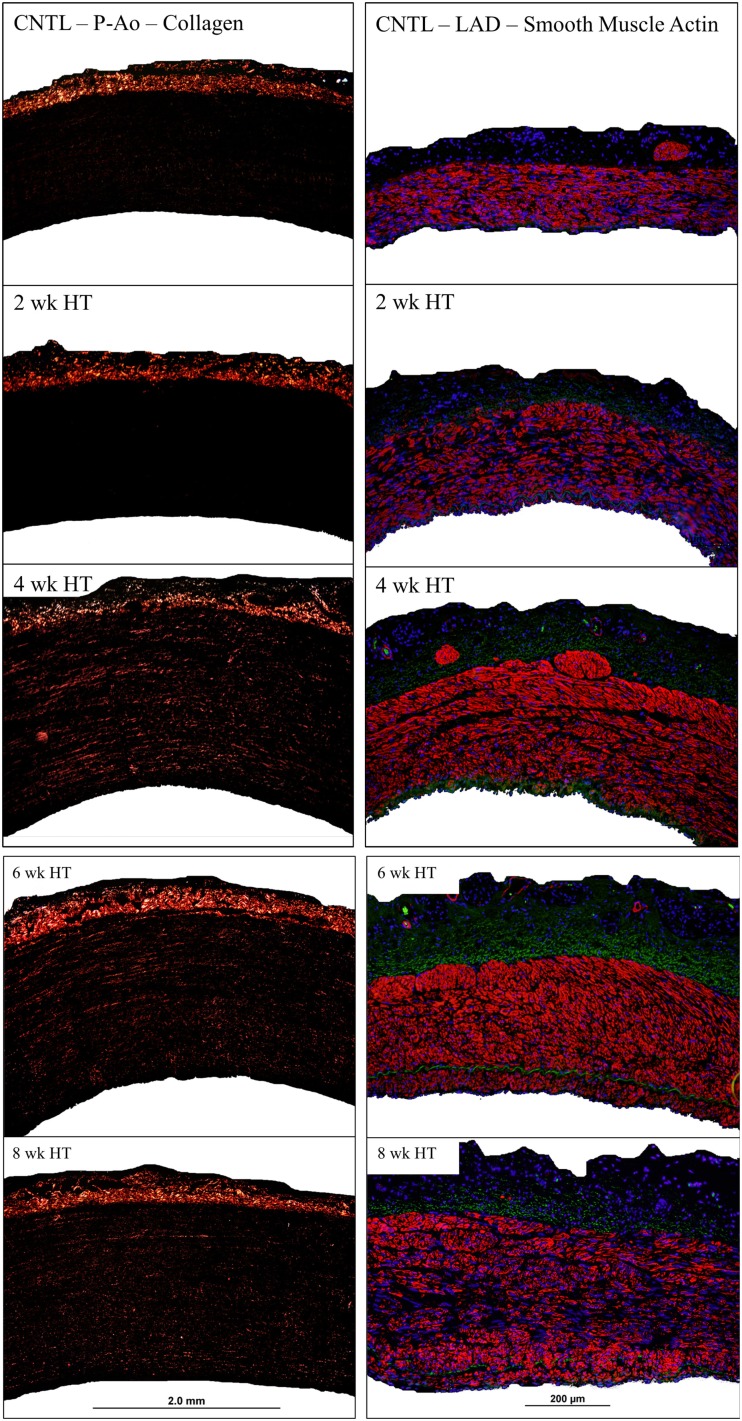
**Representative images of birefringent collagen in the P-Ao (left column) and fluorescent αSMA in the LAD (right column) at all observed time points**. Arteries have been cropped to the adventitial and luminal boarders.

The cumulative arterial responses mentioned above can be appreciated visually via representative images. For example, Figure 5 (left column) shows the marked increase in medial collagen (redish-orange) in the P-Ao at 4–8 weeks. Figure 5 (right column) shows distributions of αSMA (red), elastin (green), and nuclei (blue) in a NT control and 2, 4, 6, and 8 weeks HT LAD. Notice, in particular, the medial thickening via marked increases in smooth muscle staining as well as the development of an αSMA-rich neointima in the LAD. Albeit not shown here, it was also observed that neointimal development in the P-Ao initially consisted of αSMA-negative cells, though αSMA did increase by 8 weeks.

All arteries were stained with the hemopoietic progenitor cell marker CD34, which has also been reported to be a marker of vascular endothelial cells in small arteries (Fina et al., 1990). CD34+ cells were observed in the adventitia and media of NT P-Ao, and, to a greater extent, in HT P-Ao at 2 weeks. They were also noticed in the intima by week 6 of hypertension. CD34+ cells were also detected in the endothelium and sparsely throughout the media and adventitia in the LAD and MCA of NT and surgery control arteries (Figure 4B). The endothelial marker vWF was only expressed in the endothelium of MCA, which co-localized with CD34. Endothelial cells did not express vWF in the P-Ao and LAD, likely due to regional distributions of vWF in pigs (Rand et al., 1987), not the lack of endothelial cells. Interestingly, the endothelial cells in the LAD and MCA were not all positive for CD34 at 2 weeks of hypertension, though they again stained positive for CD34 at 4 weeks of hypertension. It has been suggested (Fina et al., 1990) that proliferating endothelial cells down-regulate CD34, hence there may have been an early shift to a more proliferative phenotype, consistent with the early increase in inner radius by the LAD. Hematopoietic/endothelial cells were also seen in the intima and adventitia of the LAD and in the intima and media of MCA. The overall density of these cells decreased in the LAD by week 6 and more so by week 8. Positive straining remained in the intima, media, and adventitia of the MCA at the 6 and 8-weeks times. Finally, the majority of neointimal cells were negative for both αSMA and CD34 staining. Counter examples were found at the 8-weeks time in the P-Ao and LAD, however. In both cases, some of the neointimal cells were positive for CD34 but not αSMA.

**Figure 6:**
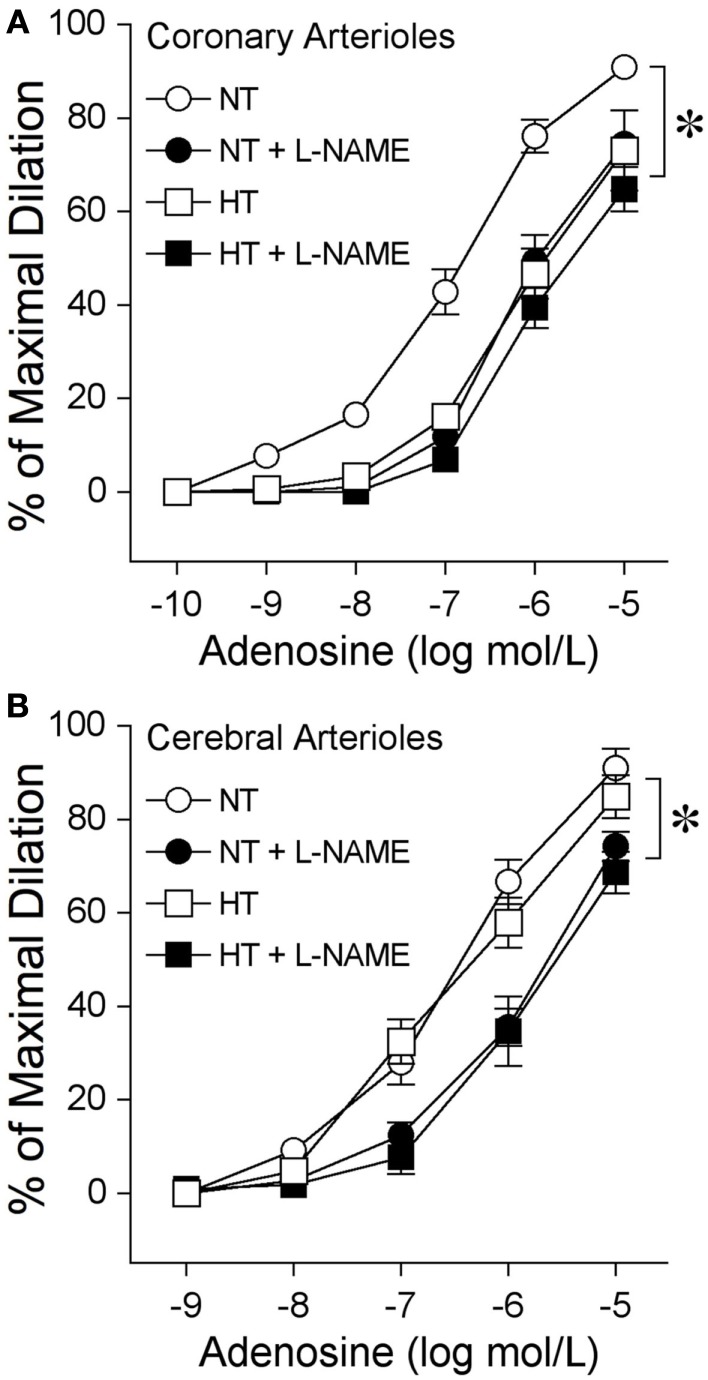
**Diminished nitric oxide-mediated dilation to adenosine at eight weeks of hypertension in coronary arterioles, but not cerebral arterioles**. **(A)** Coronary arterioles isolated from normotensive (NT) pigs dilated in a concentration-dependent manner to adenosine (*n* = 5). The NOS inhibitor l-NAME (10 μmol/l) attenuated this vasodilator response (*n* = 5). Vasodilation to adenosine (*n* = 5) was attenuated in the coronary arterioles isolated from 8-week hypertensive (HT) pigs. l-NAME did not affect this vasodilation (*n* = 5). **(B)** Cerebral arterioles isolated from NT (*n* = 5) and HT (*n* = 5) pigs dilated in a comparable manner to adenosine. l-NAME attenuated dilation of cerebral arterioles from NT (*n* = 5) and HT (*n* = 5) pigs in a comparable manner. *p < 0.05 between groups by two-way ANOVA or within groups by repeated-measures two-way ANOVA. *n* = number of vessels, excised one per animal.

The isolated and pressurized coronary arterioles from NT pigs developed basal tone (66 ± 2% of maximal diameter, with a resting diameter of 64 ± 3 μm and maximal diameter of 96 ± 4 μm; *n* = 5) within 45 min of incubation in the vessel bath. Adenosine elicited a concentration-dependent dilation of coronary arterioles from NT pigs (Figure 6A). In the presence of the NOS inhibitor l-NAME (10 μmol/l), the basal tone was not altered (63 ± 4% of maximal diameter), but dilation of these vessels to adenosine was significantly inhibited (Figure 6A). The coronary arterioles isolated from the 8-week HT pigs also developed basal tone (65 ± 1% of maximal diameter, with a resting diameter of 62 ± 3 μm and maximal diameter of 96 ± 4 μm; *n* = 5) similar to that of NT pigs, but the adenosine-elicited vasodilation was attenuated significantly (Figure 6A). In contrast to the NT coronary vessels, addition of l-NAME did not alter the dilation of HT vessels to adenosine (Figure 6A). The cerebral arterioles isolated from the NT pigs developed basal tone (45 ± 2% of maximal diameter, with a resting diameter of 39 ± 2 μm and maximal diameter of 85 ± 2 μm; *n* = 5) and dilated to adenosine in a concentration-dependent manner (Figure 6B). The cerebral arterioles isolated from the 8-week HT pigs developed a similar level of basal tone (44 ± 2% of maximal diameter, with a resting diameter of 38 ± 2 μm and maximal diameter of 83 ± 2 μm; *n* = 5) to that of NT pigs and this adenosine-elicited vasodilation was not altered by hypertension (Figure 6B). In the presence of l-NAME (10 μmol/l), the basal tone of cerebral arterioles from either NT (43 ± 3% of maximal diameter) or HT (42 ± 2% of maximal diameter) pigs was not altered, but the dilation of these vessels to adenosine was significantly inhibited in a comparable manner (Figure 6B).

## Discussion

The present results are consistent with the common observation that hypertension causes structural changes within the arterial wall, often manifesting primarily as a thickened media. Such findings were extended herein, however, by documenting differential progressive changes in elastic (proximal descending thoracic aorta) and muscular (coronary and cerebral) arteries in the same animals during the development of hypertension following the surgical creation of an aortic coarctation. Most notably, the P-Ao and LAD both thickened significantly, beginning as early as 2 weeks following the creation of the coarctation. Whereas the P-Ao thickened primarily via an increase of matrix material within the media, with some intimal thickening, the LAD thickened primarily via an increase of smooth muscle in the media, with some increases in matrix material in the adventitia. In contrast, there were no sustained, significant morphological or histological changes in the MCA up to the 8-weeks time point. Hence, the degree, type, and time course of arterial remodeling depended strongly on the location of the vessel within the arterial tree, not simply its classification as elastic or muscular.

Many animal models support the clinically observed identification of hypertension as a risk factor for atherosclerosis (Yamamoto et al., 2003; Jian-Jun and Ji-Lin, 2005). Among other factors, the role of increased macrophage activity within the wall may be central to both hypertension-induced structural changes and the associated acceleration of atherosclerosis (Maiellaro and Taylor, 2007). Angiotensin II induced hypertension in mice and rats results in a significant increase of macrophages in the adventitia and media of the aorta within 7 days (Capers et al., 1997; Carnell et al., 2007). In spontaneously HT rats, subendothelial macrophages are greatest in the carotid artery, less in the aorta, and non-existent in the renal artery (Clozel et al., 1991). In our porcine coarctation model of hypertension, we observed increases in macrophages within both the adventitial and neointimal spaces of the P-Ao and LAD starting at 2 weeks, but none in the MCA even after 8 weeks. Although not a large overall percentage, these findings suggest that monocyte invasion may occur via both the lumen and the vaso vasorum (or via peri-adventitial tissue), and may play a role in the progression of hypertension-induced remodeling of the arterial wall.

Circulating hematopoietic progenitor cells (HPCs) may localize to damaged arterial tissue through a chemotactic response. In addition, however, studies over the last decade show that the adventitia in fully developed blood vessels contains resident progenitor cells (Hu et al., 2004; Torsney et al., 2005; Passman et al., 2008). These cells are capable of migrating into the media and intima. Herein, we found CD34+ cells in both the normal and HT aortic (P-Ao) wall. Others have reported bone marrow derived progenitor cells in pulmonary arteries following either hypoxia-induced (Davie et al., 2004; Hayashida et al., 2005) or monocrotaline-induced (Spees et al., 2008) pulmonary hypertension. Colocalization of vWF+ with CD34+ markers suggests that HPCs may give rise to new endothelial cells (Wagers and Weissman, 2004; Bailey et al., 2006) or, and maybe more likely, since endothelial cells are CD34+ in NT controls, the CD34 marker is expressed in vascular endothelial cells of small arteries (Fina et al., 1990). An increased compressive radial stress, such as that experienced by arteries during hypertension, has been shown to promote the expression of smooth muscle-like proteins (αSMA and SM-MHC) in bone marrow derived stromal cells (Kobayashi et al., 2004). Likewise, we observed that HPCs in the adventitia and media of the P-Ao also co-express αSMA. Therefore, some contractile cells in the wall of HT P-Ao may have arisen from a hematopoietic origin. Yet, most HPCs in the intima, media, and adventitia of the LAD and MCA did not co-express αSMA.

We found that, for the mini-pig under NT conditions, the percentage of elastin, SMCs (indicated by αSMA), and collagen was 47.9, 25.8, and 12.8 for the P-Ao, 8.0, 29.2, and 49.0 for the LAD, and 2.8, 31.8, and 30.6 for the MCA. One can assume that the rest of the mixture constituting the wall was due primarily to proteoglycans and other cells, although αSMA stains the contractile apparatus and not the entire cell thus these SMC fractions are likely conservative. After 8 weeks of elevated blood pressure, fractions of these constituents changed to 43.8, 20.8, and 26.3 for the P-Ao, 5.0, 39.7, and 32.5 for the LAD, and 2.8, 29.8, and 36.0 for the MCA. Note, therefore, that the sum never exceeded 100% even though the measurements were independent and thus not constrained to be less than or equal to 100% (i.e., we determined collagen via PSR, elastin via VVG, and SMCs via αSMA, which required three separate samples with slightly different geometry and composition and in some cases multiple images from different regions of the wall). Knowing evolving mass fractions will be instrumental in mathematically modeling arterial behavior during hypertension-induced growth and remodeling.

The results presented herein suggest that the increased production of fibrillar collagen, often thought to be primarily responsible for the increased stiffening in hypertension and aging, depended strongly on the type of artery. The MCA and LAD had insignificant changes and significant decreases, respectively, in the percentage of collagen in hypertension whereas the relative amount of collagen increased significantly by week 4 and remained elevated in the P-Ao. It is not clear whether SMC size or number increases in preparation for or during periods of significant increases in collagen production, but cell density decreased by 5% from normal at week 2 and then increased by 8% by week 4 in the P-Ao. It may be, therefore, that cell hypertrophy preceded cell hyperplasia or nuclear ploidy. Albeit not statistically significant, there was also a slight increase (5%) in cell density in the LAD at week 2, after which the LAD did not produce proportionally more collagen. This observation may suggest that, rather than hypertrophy in preparation for matrix production, either hyperplasia or nuclear ploidy occurred first in the LAD. We suggest that prior conflicting reports as to whether hypertension first manifests as SMC hypertrophy (Olivetti et al., 1980; Owens et al., 1981; Dickhout and Lee, 2000; Hayashi and Naiki, 2009) or hyperplasia (Warshaw et al., 1979; Amann et al., 1995) may reflect, in part, differential responses in different arteries to comparable mechano-stimuli. Regardless, the present results showed that overall nuclei density remained nearly constant amid changes in thickness over the growth and remodeling time course, which suggests that there may be a preferred cell density that the artery seeks to maintain.

In addition to each type of artery having a nearly constant cell density over the growth and remodeling time course, the distribution of nuclei within the arterial wall displayed similar trends over this period. A greater nuclei density within the initial 10% of the arterial wall (see Figure A2 in Appendix, which displays nuclei density versus wall position at each artery condition) may have been due to a greater packing density in the intima, perhaps due to a greater percentage of axially oriented cells. Nevertheless, the fact that cell count remained elevated and nearly constant within the initial three quarters of the P-Ao wall, amongst the space filling elastin and glycosaminoglycans, suggested that overlapping of the concentric SMC layers may have also contributed to a higher cell count (see Figure A2 in Appendix). Unlike in the P-Ao and LAD, the transmural distribution of nuclei in the MCA appeared to change over time. This may have been due to the relatively smaller number of nuclei per MCA sample (on the order of hundreds rather than thousands for the P-Ao and LAD) and greater density (0.005497 versus 0.000127, 0.00378 cells/μm^2^).

In response to a sustained increase in pressure, it appears that the arterial wall thickens in an attempt to restore the intramural stress to homeostatic values (Humphrey, 2008; Hayashi and Naiki, 2009). Herein, both the P-Ao and LAD thickened dramatically within 2 weeks. For the P-Ao, this increase in mass was accomplished first via an increase in smooth muscle, then by a percentage increase in collagen. This finding suggests that these arteries may have first responded to the elevated blood pressure in the quickest way possible (cell hypertrophy, then hyperplasia) and then entrenched the wall at this favorable state (with hoop stress restored toward normal) by laying down new matrix material (i.e., collagen, which could also increase the stiffness). Such a scenario may be consistent with the inverse correlation proposed by Stegemann and Nerem, 2003a,b; that is, increased cell contractility (αSMA) often associates with decreased cell proliferation and vice versa. The LAD, on the other hand, increased wall thickness primarily via an increase in smooth muscle whereas the MCA did not change thickness or composition within the period of study. Knowing the time course of geometric and structural changes can obviously aid in the development of improved computational growth and remodeling models as well as contribute to improved clinical and pharmaceutical applications, particularly regarding the timing thereof. For example, in light of the results presented herein, a patient with a record of MAP above 150 mmHg for 1 month may well have a stiffer P-Ao and LAD, with increased collagen in the P-Ao and decreased collagen and increased smooth muscle in the LAD. Such differential remodeling could suggest the need for combined therapeutics to target different proteins, cell types, or intracellular pathways in different types of arteries, perhaps with the earliest focus on central arteries.

The results reported herein provide considerable new insight into important spatiotemporal variations in vascular remodeling in response to hypertension. It is important, however, to note limitations that may have affected the interpretation of these results. Notwithstanding a sample size of five to seven mini-pigs per time point, considerable inter-animal variability is to be expected – inherent differences amongst animals, surgical procedures, and evolving responses to banding. It may be for these reasons, for example, that there was a statistically significant increase in MCA thickness at week 4, but not at weeks 0, 2, or 8. Since the three primary constituents – collagen, smooth muscle, and elastin – did not change at 4 weeks of hypertension in the MCA, this increase in thickness would have been due to other cells or ground substance matrix. Indeed, compared to the P-Ao and LAD, the distribution of nuclei throughout the wall was more random for the MCA.

It is well documented that vascular endothelial cells play an important role in the maintenance and control of cardiovascular hemostasis and homeostasis by releasing myriad factors involved in regulating thrombogenesis, angiogenesis, inflammation, immune responses, vascular growth, and vasomotor activity in autocrine and paracrine manners (Rubanyi, 1993). In particular, considerable evidence implicates the metabolite adenosine as an important mediator in the regulation of coronary and cerebral blood flow during metabolic stress (O’Regan, 2005; Duncker and Bache, 2008). Activation of the endothelium-dependent NO vasodilator pathway by adenosine has been well characterized in NT porcine cerebral and coronary arterioles (Armstead, 1997; Hein and Kuo, 1999; Hein et al., 1999), but to a lesser degree and only for coronary arterioles in the case of hypertension (Zhang et al., 2004). The current results corroborate but extend previous findings that blockade of NOS inhibits adenosine-induced dilation of coronary and cerebral arterioles from NT pigs. Interestingly, we found that the NOS-mediated dilation to adenosine was attenuated in coronary, but not cerebral, arterioles in animals after 8 weeks of hypertension. It appears that endothelial dependent relaxation of smooth muscle in coronary arterioles is more susceptible to “early” HT insult and this divergent result may be due to a difference in pathways upstream or downstream from NOS activation, which can be influenced by hypertension, between coronary and cerebral arterioles. This possibility will be addressed in future studies. Collectively, therefore, our results for arteries and arterioles suggest that there is both a spatially and a temporally progressive remodeling of vessels in hypertension, which may implicate different time courses of change in local hemodynamics that may well stem from an early stiffening of the central arteries.

## Perspectives

In an aortic coarctation model of hypertension in the mini-pig, effects of increased blood pressure manifest first in central arteries and later in distal muscular arteries and arterioles, with changes occurring earlier in coronary than cerebral beds. This spatial progression of remodeling reveals a need for computational biomechanical models that can quantify the time course of changes in local, not just systemic, hemodynamics, which in turn will enable local correlations of vascular wall mechanics with the biology. Ultimately, therefore, there is a need to create computational fluid-solid-growth models that can address the hemodynamics, changes in wall structure and properties, and the biological responses.

## Conflict of Interest Statement

The authors declare that the research was conducted in the absence of any commercial or financial relationships that could be construed as a potential conflict of interest.

## Figures and Tables

**Figure A3:**
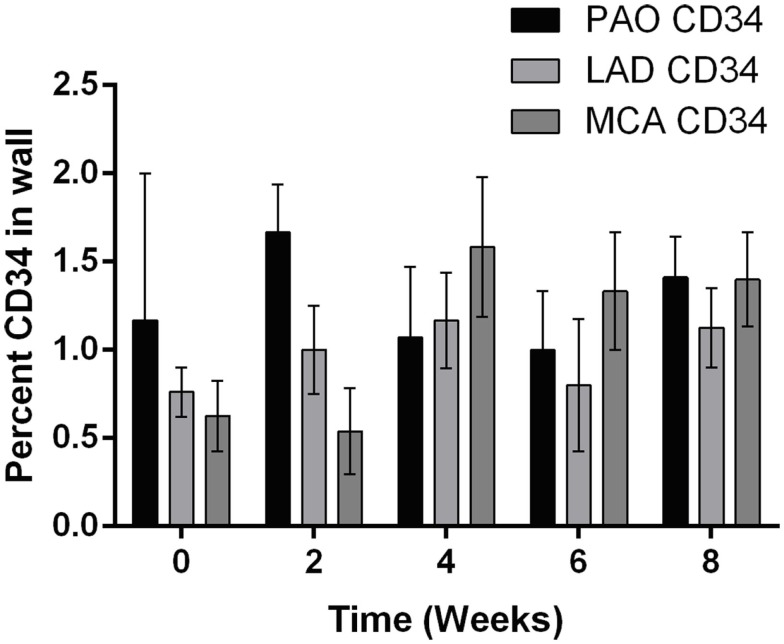
**The relative percentage of CD34, a marker for hemopeietic progenitor cells, in the P-Ao, LAD and MCA**. The change in CD34 was not statistically significant for any artery type over the 8-weeks study period as compared by two-way ANOVA with Bonferroni *post hoc* analysis.

## Appendix

**Table A1:** **Averaged number, mean arterial pressure, pulse pressure, and pulse rate of pigs at each time point after induced hypertension**.

Condition	Number of pigs	MAP averaged over the last 2 weeks (mmHg ± SD)	Pulse pressure averaged over the last 2 weeks (mmHg ± SD)	Pulse rate averaged over the last 2 weeks (bpm ± SD)
NT	16	131 ± 6	37 ± 6	34 ± 4
2 weeks HT	7	152 ± 6	48 ± 6	47 ± 6
4 weeks HT	7	171 ± 11	56 ± 12	54 ± 14
6 weeks HT	5	171 ± 14	55 ± 5	52 ± 9
8 weeks HT	6	181 ± 20	55 ± 12	54 ± 11

*Note: NT denotes normotensive and HT denotes hypertensive. MAP is mean arterial pressure and SD is standard deviation*.

**Table A2:** **Primary, secondary, and tertiary antibodies and treatment specifications for immunostaining of porcine arteries**.

Primary (clonality)	Vendor (code)	Concentration and time	Secondary	Vendor (code)	Concentration and time	Tertiary	Vendor (code)	Concentration and time
aSMA (P)	Abcam (ab5694)	2 μg/ml; RT 60 min	Alexa 594	Invitrogen (A11012)	4 μg/ml; RT 60 min			
L1 protein (M: MAC387)	Abcam (ab22506)	20 μg/ml; 4°C overnight	Alexa 488	Invitrogen (A11054)	10 μg/ml; RT 60 min	Alexa 488	Invitrogen (A11054)	10 μg/ml; RT 60 min
CD34 (M: MEC 14.7)	Abcam (ab8158)	2 μg/ml; 4°C overnight	Alexa 594	Invitrogen (A21211)	4 μg/ml; RT 60 min	Alexa 594	Invitrogen (A11012)	4 μg/ml; RT 60 min
vWF (P)	Abcam (ab6994)	80 μg/ml; RT 60 min	Alexa 594	Invitrogen (A11012)	4 μg/ml; RT 60 min			

*Note: P denotes polyclonal, M denotes monoclonal, Alexa denotes Alexa Fluor®*.

## Abbreviations

αSMA, alpha smooth muscle actin; CD34, cluster of differentiation 34 for hematopoietic stem cells; HT, hypertensive; LAD, left anterior descending artery; MAC387, macrophage marker; MCA, middle cerebral artery; NT, normotensive; PSR, picro-sirius red; P-Ao, proximal aorta; VVG, Verhoeff–van Gieson; vWF, von Willebrand factor.

## References

[B1] Abcam (2010). Ihc Paraffin Staining Protocol. Cambridge: Abcam plc

[B2] Amann K. Gharehbaghi H. Stephen S. Mall G. (1995). Hypertrophy and hyperplasia of smooth muscle cells of small intramyocardial arteries in spontaneously hypertensive rats. Hypertension 25, 124–131 10.1161/01.HYP.25.1.124 7843743

[B3] Armstead W. M. (1997). Role of nitric oxide, cyclic nucleotides, and the activation of ATP-sensitive K+ channels in the contribution of adenosine to hypoxia-induced pial artery dilation. J. Cereb. Blood Flow Metab. 17, 100–108 8978392 10.1097/00004647-199701000-00013

[B4] Bailey A. S. Willenbring H. Jiang S. Anderson D. A. Schroeder D. A. Wong M. H. (2006). Myeloid lineage progenitors give rise to vascular endothelium. Proc. Natl. Acad. Sci. U.S.A. 103, 13156–13161 10.1073/pnas.0604203103 16920790 PMC1559769

[B5] Capers Q. T. Alexander R. W. Lou P. De Leon H. Wilcox J. N. Ishizaka N. (1997). Monocyte chemoattractant protein-1 expression in aortic tissues of hypertensive rats. Hypertension 30, 1397–1402 10.1161/01.HYP.30.6.1397 9403559

[B6] Carnell P. H. Vito R. P. Taylor W. R. (2007). Characterizing intramural stress and inflammation in hypertensive arterial bifurcations. Biomech. Model. Mechanobiol. 6, 409–421 10.1007/s10237-006-0067-5 17186311

[B7] Ceravolo R. Maio R. Pujia A. Sciacqua A. Ventura G. Costa M. C. (2003). Pulse pressure and endothelial dysfunction in never-treated hypertensive patients. J. Am. Coll. Cardiol. 41, 1753–1758 10.1016/S0735-1097(03)00295-X 12767660

[B8] Clozel M. Kuhn H. Hefti F. Baumgartner H. R. (1991). Endothelial dysfunction and subendothelial monocyte macrophages in hypertension. Effect of angiotensin converting enzyme inhibition. Hypertension 18, 132–141 10.1161/01.HYP.18.4_Suppl.II37 1653185

[B9] Davidson J. M. Hill K. E. Mason M. L. Giro M. G. (1985). Longitudinal gradients of collagen and elastin gene expression in the porcine aorta. J. Biol. Chem. 260, 1901–1908 3838176

[B10] Davie N. J. Crossno J. T. Jr. Frid M. G. Hofmeister S. E. Reeves J. T. Hyde D. M. (2004). Hypoxia-induced pulmonary artery adventitial remodeling and neovascularization: contribution of progenitor cells. Am. J. Physiol. Lung Cell. Mol. Physiol. 286, L668–L678 10.1152/ajplung.00108.2003 12754186

[B11] Davis E. C. (1993). Stability of elastin in the developing mouse aorta: a quantitative radioautographic study. Histochemistry 100, 17–26 10.1007/BF00268874 8226106

[B12] Dickhout J. G. Lee R. M. (2000). Increased medial smooth muscle cell length is responsible for vascular hypertrophy in young hypertensive rats. Am. J. Physiol. Heart Circ. Physiol. 279, H2085–H2094 11045941 10.1152/ajpheart.2000.279.5.H2085

[B13] Duncker D. J. Bache R. J. (2008). Regulation of coronary blood flow during exercise. Physiol. Rev. 88, 1009–1086 10.1152/physrev.00045.2006 18626066

[B14] Fina L. Molgaard H. V. Robertson D. Bradley N. J. Monaghan P. Delia D. (1990). Expression of the CD34 gene in vascular endothelial cells. Blood 75, 2417–2426 1693532

[B15] Fossum T. W. Baltzer W. I. Miller M. W. Aguirre M. Whitlock D. Solter P. (2003). A novel aortic coarctation model for studying hypertension in the pig. J. Invest. Surg. 16, 35–44 10.1080/08941930390153032 12554338

[B16] Garcia M. Kassab G. S. (2009). Right coronary artery becomes stiffer with increase in elastin and collagen in right ventricular hypertrophy. J. Appl. Physiol. 106, 1338–1346 10.1152/japplphysiol.90592.2008 19179652 PMC2698635

[B17] Glasser S. P. Arnett D. K. (2008). Vascular stiffness and the “chicken-or-the-egg” question. Hypertension 51, 177–178 10.1161/HYPERTENSIONAHA.107.098830 18195167

[B18] Gleason R. L. Humphrey J. D. (2004). A mixture model of arterial growth and remodeling in hypertension: altered muscle tone and tissue turnover. J. Vasc. Res. 41, 352–363 10.1159/000080699 15353893

[B19] Greenwald S. E. (2007). Ageing of the conduit arteries. J. Pathol. 211, 157–172 10.1002/path.2101 17200940

[B20] Hayashi K. Naiki T. (2009). Adaptation and remodeling of vascular wall; biomechanical response to hypertension. J. Mech. Behav. Biomed. Mater. 2, 3–19 10.1016/j.jmbbm.2008.05.002 19627803

[B21] Hayashida K. Fujita J. Miyake Y. Kawada H. Ando K. Ogawa S. (2005). Bone marrow-derived cells contribute to pulmonary vascular remodeling in hypoxia-induced pulmonary hypertension. Chest 127, 1793–1798 10.1378/chest.127.5.1793 15888860

[B22] Hein T. W. Belardinelli L. Kuo L. (1999). Adenosine A2A receptors mediate coronary microvascular dilation to adenosine: role of nitric oxide and ATP-sensitive potassium channels. J. Pharmacol. Exp. Ther. 291, 655–664 10525085

[B23] Hein T. W. Kuo L. (1999). cAMP-independent dilation of coronary arterioles to adenosine: role of nitric oxide, G proteins, and K(ATP) channels. Circ. Res. 85, 634–642 10.1161/01.RES.85.7.634 10506488

[B24] Hu J. J. Fossum T. W. Miller M. W. Xu H. Liu J. C. Humphrey J. D. (2006). Biomechanics of the porcine basilar artery in hypertension. Ann. Biomed. Eng. 35, 19–29 10.1007/s10439-006-9186-5 17066325

[B25] Hu Y. Zhang Z. Torsney E. Afzal A. R. Davison F. Metzler B. (2004). Abundant progenitor cells in the adventitia contribute to atherosclerosis of vein grafts in ApoE-deficient mice. J. Clin. Invest. 113, 1258–1265 10.1172/JCI19628 15124016 PMC398426

[B26] Humphrey J. D. (2002). Cardiovascular Solid Mechanics: Cells Tissues and Organs. New York: Springer, 757

[B27] Humphrey J. D. (2008). Mechanisms of arterial remodeling in hypertension: coupled roles of wall shear and intramural stress. Hypertension 52, 195–200 10.1161/HYPERTENSIONAHA.107.103440 18541735 PMC2753501

[B28] Humphrey J. D. Na S. (2002). Elastodynamics and arterial wall stress. Ann. Biomed. Eng. 30, 509–523 10.1114/1.1467676 12086002

[B29] Jian-Jun L. Ji-Lin C. (2005). Inflammation may be a bridge connecting hypertension and atherosclerosis. Med. Hypotheses 64, 925–929 10.1016/j.mehy.2004.10.016 15780486

[B30] Kingsley K. Carroll K. Huff J. L. Plopper G. E. (2001). Photobleaching of arterial autofluorescence for immunofluorescence applications. BioTechniques 30, 794–797 11314262 10.2144/01304st05

[B31] Kobayashi N. Yasu T. Ueba H. Sata M. Hashimoto S. Kuroki M. (2004). Mechanical stress promotes the expression of smooth muscle-like properties in marrow stromal cells. Exp. Hematol. 32, 1238–1245 10.1016/j.exphem.2004.08.011 15588948

[B32] Kuo L. Chilian W. M. Davis M. J. (1991). Interaction of pressure- and flow-induced responses in porcine coronary resistance vessels. Am. J. Physiol. 261, H1706–H1715 1750529 10.1152/ajpheart.1991.261.6.H1706

[B33] Langille B. L. (1996). Arterial remodeling: relation to hemodynamics. Can. J. Physiol. Pharmacol. 74, 834–841 10.1139/y96-082 8946070

[B34] Maiellaro K. Taylor W. R. (2007). The role of the adventitia in vascular inflammation. Cardiovasc. Res. 75, 640–648 10.1016/j.cardiores.2007.06.023 17662969 PMC3263364

[B35] Miller M. J. Pinto A. Mullane K. M. (1987). Impaired endothelium-dependent relaxations in rabbits subjected to aortic coarctation hypertension. Hypertension 10, 164–170 10.1161/01.HYP.10.2.164 3112001

[B36] Monici M. (2005). Cell and tissue autofluorescence research and diagnostic applications. Biotechnol. Annu. Rev. 11, 227–256 10.1016/S1387-2656(05)11007-2 16216779

[B37] Narkiewicz K. Kjeldsen S. E. Oparil S. Hedner T. (2007). Hypertension and cardiovascular disease: is arterial stiffness the heart of the matter? Blood Press. 16, 236–237 10.1080/08038020701561687 17917863

[B38] Olivetti G. Anversa P. Melissari M. Loud A. V. (1980). Morphometry of medial hypertrophy in the rat thoracic aorta. Lab. Invest. 42, 559–565 7382430

[B39] O’Regan M. (2005). Adenosine and the regulation of cerebral blood flow. Neurol. Res. 27, 175–181 10.1179/016164105X21931 15829181

[B40] O’Rourke M. F. Nichols W. W. (2005). Aortic diameter, aortic stiffness, and wave reflection increase with age and isolated systolic hypertension. Hypertension 45, 652–658 10.1161/01.HYP.0000153793.84859.b8 15699456

[B41] Owens G. K. Rabinovitch P. S. Schwartz S. M. (1981). Smooth muscle cell hypertrophy versus hyperplasia in hypertension. Proc. Natl. Acad. Sci. U.S.A. 78, 7759–7763 10.1073/pnas.78.12.7759 6950415 PMC349350

[B42] Passman J. N. Dong X. R. Wu S. P. Maguire C. T. Hogan K. A. Bautch V. L. (2008). A sonic hedgehog signaling domain in the arterial adventitia supports resident Sca1+ smooth muscle progenitor cells. Proc. Natl. Acad. Sci. U.S.A. 105, 9349–9354 10.1073/pnas.0711382105 18591670 PMC2453724

[B43] Payne R. A. Webb D. J. (2006). Arterial blood pressure and stiffness in hypertension: is arterial structure important? Hypertension 48, 366–367 10.1161/01.HYP.0000237668.31786.1f 16908759

[B44] Rand J. H. Badimon L. Gordon R. E. Uson R. R. Fuster V. (1987). Distribution of von Willebrand factor in porcine intima varies with blood vessel type and location. Arteriosclerosis 7, 287–291 10.1161/01.ATV.7.3.287 3297010

[B45] Rizvi M. A. Katwa L. Spadone D. P. Myers P. R. (1996). The effects of endothelin-1 on collagen type I and type III synthesis in cultured porcine coronary artery vascular smooth muscle cells. J. Mol. Cell. Cardiol. 28, 243–252 10.1006/jmcc.1996.0023 8729057

[B46] Rizvi M. A. Myers P. R. (1997). Nitric oxide modulates basal and endothelin-induced coronary artery vascular smooth muscle cell proliferation and collagen levels. J. Mol. Cell. Cardiol. 29, 1779–1789 10.1006/jmcc.1996.0480 9236133

[B47] Rubanyi G. M. (1993). The role of endothelium in cardiovascular homeostasis and diseases. J. Cardiovasc. Pharmacol. 22(Suppl. 4), S1–S14 10.1097/00005344-199322002-00002 7523767

[B48] Shapiro S. D. Endicott S. K. Province M. A. Pierce J. A. Campbell E. J. (1991). Marked longevity of human lung parenchymal elastic fibers deduced from prevalence of d-aspartate and nuclear weapons-related radiocarbon. J. Clin. Invest. 87, 1828–1834 10.1172/JCI115215 2022748 PMC295305

[B49] Spees J. L. Whitney M. J. Sullivan D. E. Lasky J. A. Laboy M. Ylostalo J. (2008). Bone marrow progenitor cells contribute to repair and remodeling of the lung and heart in a rat model of progressive pulmonary hypertension. FASEB J. 22, 1226–1236 10.1096/fj.07-8076com 18032636

[B50] Stegemann J. P. Nerem R. M. (2003a). Altered response of vascular smooth muscle cells to exogenous biochemical stimulation in two- and three-dimensional culture. Exp. Cell Res. 283, 146–155 10.1016/S0014-4827(02)00041-1 12581735

[B51] Stegemann J. P. Nerem R. M. (2003b). Phenotype modulation in vascular tissue engineering using biochemical and mechanical stimulation. Ann. Biomed. Eng. 31, 391–402 10.1114/1.1558031 12723680

[B52] Tang E. H. Vanhoutte P. M. (2010). Endothelial dysfunction: a strategic target in the treatment of hypertension? Pflugers Arch. 459, 995–1004 10.1007/s00424-010-0786-4 20127126

[B53] Torsney E. Hu Y. Xu Q. (2005). Adventitial progenitor cells contribute to arteriosclerosis. Trends Cardiovasc. Med. 15, 64–68 10.1016/j.tcm.2005.02.003 15885572

[B54] Valentin A. Cardamone L. Baek S. Humphrey J. D. (2009). Complementary vasoactivity and matrix remodelling in arterial adaptations to altered flow and pressure. J. R. Soc. Interface 6, 293–306 10.1098/rsif.2008.0254 18647735 PMC2659584

[B55] Wagers A. J. Weissman I. L. (2004). Plasticity of adult stem cells. Cell 116, 639–648 10.1016/S0092-8674(04)00208-9 15006347

[B56] Warshaw D. M. Mulvany M. J. Halpern W. (1979). Mechanical and morphological properties of arterial resistance vessels in young and old spontaneously hypertensive rats. Circ. Res. 45, 250–259 10.1161/01.RES.45.2.250 445708

[B57] Yamamoto K. Ikeda U. Shimada K. (2003). Role of mechanical stress in monocytes/macrophages: implications for atherosclerosis. Curr. Vasc. Pharmacol. 1, 315–319 10.2174/1570161033476565 15320477

[B58] Zhang C. Hein T. W. Wang W. Miller M. W. Fossum T. W. McDonald M. M. (2004). Upregulation of vascular arginase in hypertension decreases nitric oxide-mediated dilation of coronary arterioles. Hypertension 44, 935–943 10.1161/01.HYP.0000146907.82869.f2 15492130

